# Bufalin-Loaded Multifunctional Nanodrugs for Cancer Therapy: Mechanisms, Delivery Strategies, and Translational Perspectives

**DOI:** 10.3390/biom16091297

**Published:** 2026-09-08

**Authors:** Yanrui Yang, Xinyue Zeng, Yuqiao Hu, Yufei Su, Chengqi Li, Fajin Lv, Kehui Zhao, Jing Hu

**Affiliations:** 1 Chinese Medicine Germplasm Resources Innovation and Effective Uses Key Laboratory of Sichuan Province, State Key Laboratory of Southwest Specialty Chinese Medicine Resources, College of Pharmacy, Chengdu University of Traditional Chinese Medicine, Chengdu 611137, China; 15756326127@163.com (Y.Y.); 18402896743@163.com (Y.H.); ccp182@163.com (K.Z.); 2 CDUTCM-KEELE Joint Health and Medical Sciences Institute, Chengdu University of Traditional Chinese Medicine, Chengdu 611137, China; m17348128660@163.com; 3 School of Pharmacy, Shanghai University of Traditional Chinese Medicine, Shanghai 201203, China; 4 Chinese Medicine Germplasm Resources Innovation and Effective Uses Key Laboratory of Sichuan Province, School of Modern Chinese Medicine Industry, Chengdu University of Traditional Chinese Medicine, Chengdu 611137, China; 18382117046@163.com (Y.S.); 17680736075@163.com (C.L.); 5 College of Biomedical Engineering, Chongqing Medical University, Chongqing 400016, China; anychina01@126.com

**Keywords:** bufalin, nanomedicine, drug delivery, cancer treatment

## Abstract

Bufalin is a naturally occurring bufadienolide with broad-spectrum anticancer activity. Unlike conventional cytotoxic agents, bufalin exerts pleiotropic antitumour effects by directly modulating oncogenic proteins and promoting their degradation. It also disrupts metabolic plasticity, induces multiple forms of regulated cell death, counteracts therapeutic resistance, and remodels the immunosuppressive tumour microenvironment. However, the further application of bufalin is hindered by poor aqueous solubility, rapid systemic clearance, a narrow therapeutic window, and dose-limiting toxicity. Nanodrug delivery systems (NDDSs) offer a promising strategy for translating these interconnected pharmacological effects into spatially and temporally controlled therapeutic responses. This review summarises the distinctive anticancer mechanisms of bufalin and systematically evaluates the organic, inorganic, biomimetic, and hybrid nanocarriers developed for its delivery. Particular attention is given to tumour-responsive drug release, the targeting of cancer stem cells, the induction of ferroptosis and pyroptosis, metabolic modulation, sensitisation to phototherapy, and the activation of antitumour immunity. Finally, the major translational challenges are critically examined to inform the further rational development of bufalin-based nanomedicines.

## 1. Introduction

Natural products remain an important source of anticancer agents because their structurally diverse bioactive constituents can simultaneously modulate interconnected oncogenic pathways that are difficult to target effectively with single-target therapies [1,2]. Bufalin, a C-24 bufadienolide derived from *Venenum Bufonis*, has attracted increasing attention because of its potent inhibitory effects on tumour growth, therapeutic resistance, metastatic dissemination, metabolic adaptation, and immune evasion [3,4]. Unlike conventional chemotherapeutic agents, bufalin does not exert its antitumour effects solely through nonspecific cytotoxicity. Bufalin can directly interact with molecular targets including Na^+^/K^+^-ATPase, steroid receptor coactivators family coactivators, epidermal growth factor receptor (EGFR), calcium/calmodulin-dependent protein kinase kinase 2 (CAMKK2), bifunctional apoptosis regulator (BFAR), serine/threonine kinase 33 (STK33), and hormone receptors. It also promotes the ubiquitination and subsequent degradation of selected oncogenic proteins [5]. These target-directed activities are integrated with mitochondrial dysfunction, oxidative stress, metabolic suppression, the induction of apoptosis and ferroptosis, and remodelling of the tumour microenvironment [6,7,8,9]. Therefore, these mechanisms provide a pharmacological basis for the broad-spectrum anticancer activity of bufalin.

Nevertheless, the therapeutic application of free bufalin is constrained by its high lipophilicity, poor aqueous solubility, rapid metabolism and systemic clearance, insufficient tumour exposure, narrow therapeutic window, and potential cardiotoxicity. Consequently, conventional formulations and administration routes often fail to maintain therapeutically effective intratumoural concentrations without increasing systemic exposure and toxicity [10,11].

Nanodrug delivery systems (NDDSs) provide a rational strategy for addressing these pharmacological and formulation-related limitations. Liposomes, micelles, polymeric nanoparticles, mesoporous silica nanoparticles, cell-membrane-coated nanosystems, hydrogels, carbon-based nanomaterials, and multicomponent hybrid platforms have been developed to improve bufalin loading efficiency, pharmacokinetic behaviour, tumour accumulation, and spatiotemporally controlled release [12,13,14,15,16]. More importantly, functional nanomaterials may act synergistically with bufalin by releasing bioactive ions, generating heat or reactive oxygen species, modulating tumour metabolism, inducing immunogenic cell death, or delivering immunomodulatory cargos [17,18,19,20].

Herein, we summarise the latest trends of bufalin-loaded nanodelivery systems for cancer therapy. First, we provide a comprehensive summary of unique anticancer mechanisms of bufalin. More remarkably, we delve into the integration strategies leveraging bufalin-loaded nanodelivery technologies for combating cancer, highlighting nanosystem construction, targeted delivery, drug release, and anti-cancer activities. Finally, we discuss the future prospects and challenges towards clinical translation for bufalin-based nanomedicine in cancer therapy. Through the comprehensive analysis of the integration of bufalin and various nanodelivery systems, this review will promote the combined treatment of tumours.

## 2. Methodology

We searched English databases between 2023 and 2026, including PubMed, Web of Science, and Google Scholar, and then we screened the relevant literature published in China and abroad. The databases were searched using the following terms: [“nanomedicine” OR “organic” OR “inorganic” OR “biomimetic” AND “bufalin”]. Bufalin-based nanodrug delivery systems were retrieved in the databases using the following terms: [‘‘liposome” AND ‘‘cancer”]. According to the situation of different databases, the subject words were comprehensively searched in combination with keywords, topics, abstracts, and free words to ensure the systematization and integrity of literature retrieval.

We searched all preclinical studies on bufalin-loaded multifunctional nanodrugs for cancer therapy. To ensure the authenticity and systematicness of the results, we included relevant in vitro and in vivo studies involving cell and animal models.

## 3. Unique Anticancer Mechanisms of Bufalin and Therapeutic Potential of Its Derivatives

**Figure 1:**
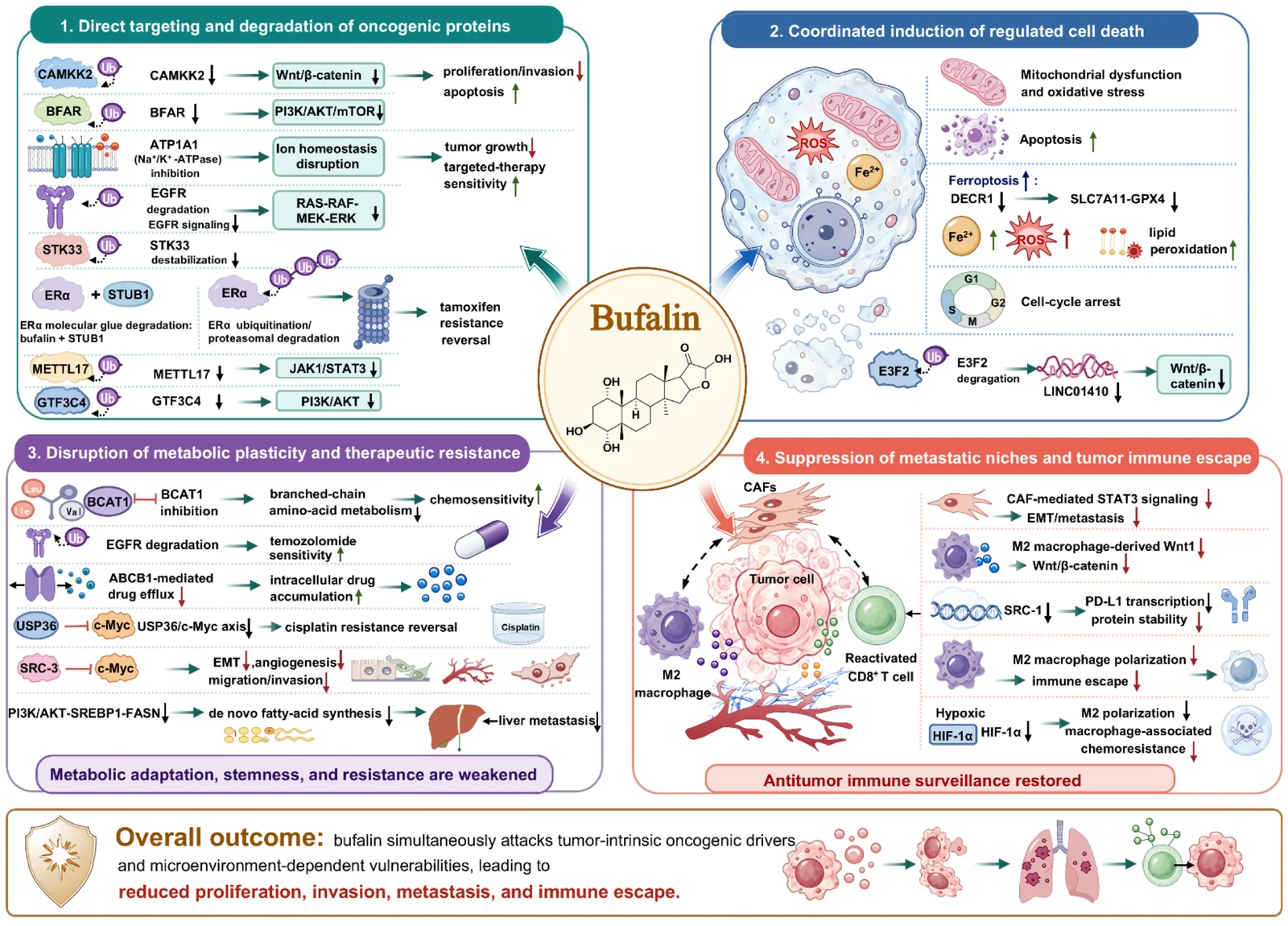
The multiple core anti-cancer mechanisms of bufalin.

The anticancer activity of bufalin is characterised by an unusually broad yet mechanistically interconnected pharmacological profile. Rather than exerting its effects solely through nonspecific cytotoxicity, bufalin can directly interact with oncogenic proteins, modulate protein stability, disrupt metabolic adaptation, activate multiple regulated cell death pathways, suppress metastatic plasticity, and restore antitumour immune surveillance. Integrated molecular and metabolomic analyses indicate that bufalin simultaneously perturbs cancer-associated signalling networks, amino acid and lipid metabolism, redox homeostasis, and cellular energy production. In parallel, inhibition of Hippo-YAP signalling further supports the ability of bufalin to restrain tumour growth by disrupting core transcriptional programmes that sustain malignant progression [21,22]. Figure 1 displays the multiple core anti-cancer mechanisms of bufalin.

### 3.1. Direct Targeting and Degradation of Oncogenic Proteins

A notable feature of bufalin is its ability to directly interact with functionally important cancer-associated proteins. In intrahepatic cholangiocarcinoma, bufalin directly binds to Ca^2+^/calmodulin-dependent protein kinase 2 and subsequently inhibits downstream Wnt/β-catenin signalling. This interaction suppresses tumour-cell proliferation and invasion while promoting apoptosis. In gastric cancer, BFAR has been identified as another direct molecular target of bufalin. By targeting BFAR, bufalin suppresses PI3K/AKT/mTOR signalling and thereby inhibits tumour growth and metastatic progression [23,24].

Na^+^/K^+^-ATPase is a well-established and pharmacologically relevant target of bufalin. Inhibition of the catalytic α1 subunit, ATP1A1, disrupts intracellular ion homeostasis and compromises melanoma-cell survival. Notably, inhibition of ATP1A1 can also restore sensitivity to targeted therapies, indicating that the pharmacological effects of bufalin on Na^+^/K^+^-ATPase extend beyond conventional ion-pump inhibition. More recent studies have broadened the molecular target profile of bufalin by showing that it promotes EGFR degradation and directly binds to STK33. Destabilisation or functional inhibition of these targets suppresses EGFR-dependent mitogenic signalling and STK33-driven progression of triple-negative breast cancer, respectively [25,26,27].

Bufalin can also function as a molecular glue, representing a relatively uncommon mechanism among natural anticancer compounds. Recent advances in targeted protein degradation have highlighted molecular glues as a powerful strategy to induce selective elimination of disease-associated proteins [28]. In oestrogen receptor-positive (ER-positive) breast cancer, bufalin was identified as an ERα-directed molecular glue that directly binds ERα and promotes its interaction with the E3 ubiquitin ligase STUB1, resulting in ERα ubiquitination and proteasomal degradation [29]. This mechanism suppresses oestrogen-dependent signalling and restores tamoxifen sensitivity in breast cancer cell lines, xenograft models, and patient-derived organoids [30].

Collectively, these findings position bufalin as more than a conventional signalling-pathway inhibitor. By engaging multiple oncogenic proteins and, in specific contexts, promoting their degradation, bufalin may achieve sustained depletion rather than transient inhibition of key molecular drivers of malignancy.

### 3.2. Coordinated Induction of Regulated Cell Death

Bufalin can activate multiple forms of regulated cell death by simultaneously disrupting mitochondrial function, redox homeostasis, oncogenic survival signalling, and cell-cycle progression. Among these mechanisms, ferroptosis has emerged as a recently recognised form of bufalin-induced regulated cell death. In breast cancer, bufalin suppresses DECR1, thereby impairing the SLC7A11-GPX4 antioxidant axis. This effect increases intracellular Fe^2+^ levels, promotes the accumulation of reactive oxygen species, and enhances lipid peroxidation, ultimately triggering ferroptotic cell death. In head and neck cancer, bufalin exacerbates mitochondrial stress, induces apoptosis and cell-cycle arrest, and suppresses β-catenin-dependent survival signalling. These effects demonstrate its ability to coordinate multiple cytotoxic responses within a single tumour context [31,32].

Bufalin-induced apoptosis is frequently associated with the disruption of oncogenic survival networks. In hepatocellular carcinoma, bufalin suppresses tumour-cell proliferation, induces apoptosis, and inhibits tumour growth by attenuating EGFR phosphorylation and the downstream RAS-RAF-MEK-ERK signalling cascade. In oesophageal squamous cell carcinoma, bufalin functions as a molecular glue that promotes E2F2 degradation and reduces the transcription of the oncogenic long non-coding RNA LINC01410. This mechanism subsequently suppresses Wnt/β-catenin signalling, thereby markedly reducing tumour-cell viability and invasive capacity [33,34].

The distinctive advantage of bufalin therefore lies not in its activation of a single cell-death pathway but in its ability to simultaneously disrupt multiple complementary survival mechanisms. Mitochondrial dysfunction, excessive oxidative stress, ferroptotic lipid damage, cell-cycle arrest, and oncogenic protein degradation may converge to limit the ability of cancer cells to compensate for therapeutic stress.

### 3.3. Disruption of Metabolic Plasticity and Reversal of Therapeutic Resistance

Cancer cells depend on metabolic plasticity to sustain proliferation and survive anticancer treatment. Bufalin can directly disrupt this adaptive capacity. In pancreatic cancer, branched-chain amino acid transaminase 1 (BCAT1) has been identified as a functional target of bufalin. Inhibition of BCAT1 disrupts branched-chain amino acid metabolism, reduces cellular metabolic fitness, and enhances sensitivity to chemotherapeutic agents. Bufalin induces autophagic degradation of EGFR via chaperones in glioblastoma and sensitizes to temozolomide cytotoxicity. This finding indicates that modulation of protein turnover may be an effective approach to overcome therapeutic resistance [35,36].

Bufalin also bypasses drug transporter- and deubiquitinase-mediated resistance mechanisms. Bufalin inhibited the ABCB1 efflux pump in docetaxel-resistant breast cancer cells, restoring the intracellular accumulation of drugs and resensitising the cells to chemotherapy. Bufalin inhibits the USP36/c-Myc axis in cisplatin-resistant ovarian cancer. Inhibition of USP36 destabilizes c-Myc, decreases its oncogenic transcriptional activity and restores cisplatin sensitivity in cellular and animal models [37,38].

The metabolic effects of bufalin are closely linked to its antimetastatic activity. In chemotherapy-resistant colorectal cancer, bufalin suppresses the SRC-3/c-Myc pathway, counteracts resistance-associated epithelial–mesenchymal transition, and inhibits angiogenesis, migration, and invasion. Bufalin also inhibits de novo fatty acid synthesis by suppressing the PI3K/AKT/SREBP1/FASN signalling axis. Because fatty acid synthesis provides the membrane components, signalling lipids, and energy substrates required for metastatic colonisation, disruption of this pathway markedly suppresses liver metastasis from colorectal cancer [39,40].

Thus, bufalin may overcome therapeutic resistance at multiple mechanistic levels, including drug efflux, oncogenic protein stabilisation, compensatory metabolic reprogramming, and resistance-associated epithelial–mesenchymal transition. This multilevel activity is particularly valuable because therapeutic resistance is rarely driven by a single molecular alteration.

### 3.4. Suppression of Metastatic Niches and Tumor Immune Escape

Bufalin also exerts substantial effects on the non-malignant cellular components of the tumour microenvironment that support tumour progression. Cancer-associated fibroblasts promote tumour invasion and metastasis through cytokine secretion, extracellular matrix remodelling, and sustained signal transducer and activator of transcription 3 (STAT3) signalling. Bufalin suppresses fibroblast-induced STAT3 activation in colorectal cancer cells, thereby inhibiting epithelial–mesenchymal transition and metastatic dissemination. In hepatocellular carcinoma, bufalin reduces Wnt1 production by M2-polarised tumour-associated macrophages, consequently attenuating Wnt/β-catenin signalling in malignant hepatocytes. This dual action attenuates both the protumour macrophage phenotype and macrophage-to-tumour-cell signalling [41,42].

Bufalin also modulates immune-checkpoint signalling and macrophage-associated immune-evasion programmes. In colorectal cancer cells, steroid receptor coactivator-1 (SRC-1) enhances programmed death-ligand 1 (PD-L1) transcription and promotes PD-L1 protein stability. By targeting SRC-1, bufalin suppresses PD-L1 expression and thereby enhances antitumour immune activity. In metastatic colorectal cancer, bufalin inhibits M2 macrophage polarisation, thereby alleviating macrophage-mediated immunosuppression and limiting immune evasion and metastatic progression. Furthermore, under the hypoxic conditions associated with chemotherapy-resistant colorectal cancer, bufalin suppresses the steroid receptor coactivator-3 (SRC-3)/hypoxia-inducible factor 1α (HIF-1α) axis and inhibits M2 macrophage polarisation, thereby counteracting macrophage-associated chemoresistance [43,44,45].

In summary, these findings indicate that bufalin acts across multiple interconnected layers of cancer biology. It promotes the degradation of selected oncogenic proteins, activates complementary forms of regulated cell death, disrupts metabolic adaptation and therapeutic resistance, suppresses prometastatic signalling, and remodels the immunosuppressive tumour microenvironment. This capacity to simultaneously target tumour-intrinsic and microenvironment-dependent vulnerabilities represents a key pharmacological advantage of bufalin as an anticancer agent.

### 3.5. Bufalin Derivatives as Potential Anticancer Agents

In addition to studies of the parent compound, medicinal-chemistry optimisation of the bufadienolide scaffold has been explored as a complementary strategy to improve anticancer potency, physicochemical properties, target selectivity, and therapeutic safety. Structural modification and structure–activity relationship studies of bufadienolides have identified the C3 substituent and the C17 unsaturated lactone moiety as important determinants of biological activity [46,47,48,49]. Early structure–activity studies identified the C3 position of bufalin as a site amenable to chemical modification. A series of nitrogen-containing bufalin-3-yl carbamate derivatives showed potent antiproliferative activity, with several representative compounds also demonstrating marked antitumour effects in vitro and in vivo [50]. Among these compounds, BF211 has received the most extensive investigation. Compared with bufalin, BF211 induced greater apoptosis in A549 lung cancer cells while showing lower acute toxicity in mice and significant antitumour activity in xenograft models [51]. Mechanistic studies showed that BF211 inhibited proteasome activity by reducing expression of the proteasome β1 subunit and disrupting 26S proteasome assembly [52]. In multiple myeloma, BF211 also promoted apoptotic cell death through suppression of the IL-6/JAK2/STAT3 pathway [53]. Chemical derivatisation has also been investigated as a means of improving tumour selectivity. The FAPα-responsive prodrug BF211-03 was designed to preferentially release BF211 in FAPα-rich tumour environments. In HCT116 xenograft models, it retained antitumour efficacy while reducing treatment-related systemic toxicity [54]. More recent SAR studies suggest that successful structural modification depends on preserving key pharmacophoric features. Methylation of the native C3β-hydroxyl group, or replacement of the six-membered unsaturated lactone at C17 with a five-membered lactone, markedly reduced antiproliferative activity. These findings support the importance of the C3β configuration and the native C17 lactone scaffold [55]. Recent integrated evaluations of bufalin derivatives similarly support rational modification at C3 but also point to the need to balance cytotoxic potency with physicochemical and pharmacological properties [56]. Taken together, these findings suggest that bufalin derivatisation serves purposes beyond simply increasing cytotoxicity. Rational structural optimisation may also alter target engagement, tumour selectivity, pharmacological potency, and systemic toxicity, offering a medicinal-chemistry strategy that complements nanodelivery approaches for improving the therapeutic potential of bufalin.

Overall, current evidence suggests that bufalin acts at several interconnected levels of cancer biology. It promotes the degradation of selected oncogenic proteins, activates multiple forms of regulated cell death, interferes with metabolic adaptation and therapeutic resistance, suppresses prometastatic signalling, and alters the immunosuppressive tumour microenvironment. The ability to act on both tumour-intrinsic and microenvironment-dependent vulnerabilities may therefore be an important pharmacological feature of bufalin. Studies of bufalin derivatives further suggest that rational structural modification may preserve or enhance these anticancer properties while also modifying pharmacological potency, tumour selectivity, and systemic toxicity. Together, the pleiotropic pharmacology of bufalin and the medicinal-chemistry optimisation of its derivatives provide a rationale for the continued development of bufalin-based anticancer therapeutics.

## 4. Application of Bufalin-Based Nanodelivery System in Cancer Treatment

### 4.1. Organic Nanocarrier

Liposomes, polymeric nanoparticles, micelles, dendrimers and lipid-polymer hybrid nanoparticles are some of the most widely studied platforms for anticancer drug delivery [57,58,59] and are termed as organic nanocarriers. These systems are generally formulated from biodegradable organic materials and offer versatile platforms to enhance the aqueous solubility, pharmacokinetic behaviour, tumour accumulation and controlled release of hydrophobic anticancer drugs [60,61]. Such limitations can be overcome by organic nanocarriers through efficient drug encapsulation, surface functionalization with targeting ligands, drug release in response to the tumour microenvironment and co-delivery of complementary therapeutic agents [62,63]. Therefore, organic nanoplatforms are a reasonable strategy to enhance the delivery of bufalin and broaden its application in cancer therapy.

**Figure 2:**
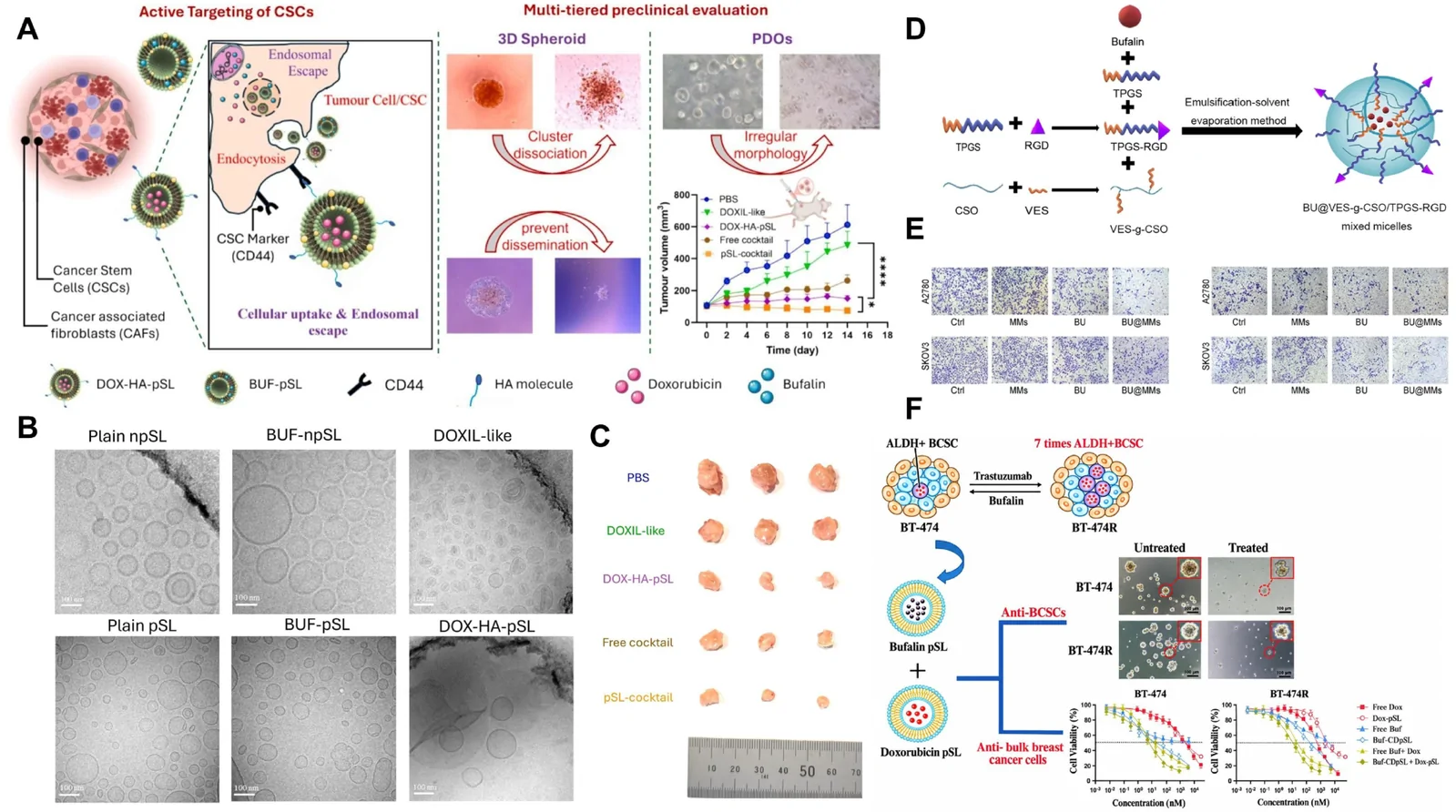
Application of organic nanocarriers loaded with bufalin in cancer therapy. (**A**) Schematic diagram of the composition of DOX-HA-pSL as well as its therapeutic mechanism. (**B**) Cryo-TEM images of various liposomes containing DOX or Buf. (**C**) In vivo therapeutic evaluation of pSL-cocktail versus control formulations in an HCC1954 orthotopic xenograft model: Gross morphology of excised tumours at endpoint. Reproduced from reference [72]. Copyright 2026, Elsevier Ltd. (**D**) BU@MMs formulation mechanism. (**E**) Representative images of the migrating and invasion of A2780 and SKOV3 cells. Reproduced from reference [80]. Copyright 2023, Springer Nature. (**F**) Schematic diagram of the therapeutic mechanism of bufalin-pSL collaborates with Doxorubicin-pSL. Reproduced from reference [88]. Copyright 2024, Taylor & Francis.

Liposomal nanococktails are multifunctional liposome-based co-delivery systems designed to encapsulate multiple therapeutic agents within a single vesicle, with the aim of producing complementary or synergistic anticancer effects [64,65,66]. Compared with conventional single-drug liposomes, dual-drug liposomes are distinguished mainly by the simultaneous delivery of two or more therapeutic agents with different or complementary mechanisms of action rather than by the composition of the carrier itself. These systems retain the main advantages of conventional liposomes, including high biocompatibility, high drug-loading capacity, and tunable membrane properties [67]. The phospholipid bilayer and aqueous core allow hydrophobic and hydrophilic agents to be incorporated at the same time. The formulation can also be engineered for stimuli-responsive drug release [68,69]. Liposomal nanococktails are usually composed of phospholipids together with auxiliary components such as dioleoyl phosphatidylethanolamine (DOPE), phosphatidylcholine derivatives, cholesterol, PEGylated lipids, and targeting ligands. This multifunctional co-delivery approach allows different therapeutic agents to be delivered in a coordinated manner and may improve anticancer efficacy through additive or synergistic effects [70,71]. Ahmed et al. [72] developed a pH-sensitive fusogenic liposomal nanococktail for co-delivery of bufalin and doxorubicin (DOX) (Figure 2A). The formulation contained DOPE, HSPC or DSPC, CHEMS, DSPE-PEG_2000_ and hyaluronic acid. The engineered nanoplatform was stable under physiological conditions but acid-destabilised in the acidic conditions of the tumour microenvironment, allowing tumour penetration and intracellular drug release (Figure 2B). Co-delivery of bufalin and DOX combination exhibited synergistic anti-tumour activity against human epidermal growth factor receptor 2 (HER2)-positive breast cancer by simultaneously targeting the proliferating tumour cells and cancer stem cell populations. Mechanistically, the nanococktail impaired mammosphere formation, self-renewal, and the invasive capacity of cancer stem cells, resulting in marked tumour regression in xenograft and patient-derived tumour models (Figure 2C).

Polymeric micelles are nanoscale self-assembled carriers composed of amphiphilic polymers or lipid-polymer conjugates [73,74]. They typically comprise a hydrophobic core for drug encapsulation and a hydrophilic corona that promotes aqueous dispersibility and colloidal stability under physiological conditions [75]. These nanostructures are commonly constructed from biodegradable polymers and functional materials, including chitosan, poly(lactic-co-glycolic acid) (PLGA), polyethylene glycol (PEG), hyaluronic acid, and vitamin E derivatives [76,77]. Their composition can be tailored to achieve efficient drug loading, prolonged systemic circulation, tumour-targeted delivery, and controlled intracellular release [78,79]. Xu et al. [80] developed bufalin-loaded mixed micelles composed of vitamin E succinate-grafted chitosan oligosaccharide and RGD-conjugated TPGS for the targeted treatment of ovarian cancer (Figure 2D). In this system, the amphiphilic architecture formed by vitamin E succinate-grafted chitosan oligosaccharide and RGD-functionalised TPGS enhanced bufalin encapsulation and formulation stability (Figure 2E). Meanwhile, RGD-mediated recognition of integrin αvβ3 facilitated the preferential uptake of the micelles by ovarian cancer cells overexpressing this receptor. The micelles markedly reduced intraperitoneal tumour dissemination by inhibiting epithelial–mesenchymal transition. This effect was accompanied by increased E-cadherin expression and decreased expression of N-cadherin, vimentin, Snail, matrix metalloproteinase-2 (MMP-2), matrix metalloproteinase-9 (MMP-9), and vascular endothelial growth factor (VEGF).

Liposomes are spherical vesicular nanocarriers composed of one or more phospholipid bilayers enclosing an aqueous core [81,82,83]. Hydrophilic agents can be encapsulated within the aqueous compartment, whereas hydrophobic agents partition into the lipid bilayer. Owing to their favourable biocompatibility, biodegradability, and compositional versatility, liposomes are widely used as drug-delivery vehicles [84,85,86]. They are commonly formulated using phospholipids and auxiliary lipids, including DOPE, phosphatidylcholine derivatives, cholesterol, and PEGylated lipids, which collectively regulate membrane stability, circulation behaviour, drug retention, and stimuli-responsive release [87]. Gao et al. [88] developed a pH-sensitive liposomal platform for the co-delivery of bufalin and doxorubicin to treat trastuzumab-resistant HER2-positive breast cancer (Figure 2F). In this system, bufalin was incorporated as a cancer stem cell (CSC)-targeting agent, whereas doxorubicin was used to eliminate the bulk tumour-cell population. The pH-responsive liposomes were formulated using a DOPE/CHEMS-based lipid composition. Under acidic conditions, protonation of CHEMS destabilised the lipid bilayer and promoted intracellular drug release. The liposomal co-delivery of bufalin and doxorubicin produced synergistic antitumour effects by concurrently targeting breast cancer stem cells and proliferating tumour cells. In particular, the formulation reduced the proportion of ALDH-positive breast cancer stem cells by approximately 85%, suppressed tumour-sphere formation and self-renewal, and enhanced cytotoxicity against both trastuzumab-sensitive and trastuzumab-resistant HER2-positive breast cancer cells.

In summary, organic nanocarriers provide versatile platforms for improving the aqueous solubility, tumour accumulation, and therapeutic exposure of bufalin. Micelles, liposomes, and lipid-based nanococktails can further enable active targeting, stimuli-responsive release, and combination therapy, thereby strengthening the ability of bufalin to suppress metastatic dissemination, cancer stem cell activity, and the progression of drug-resistant tumours.

### 4.2. Inorganic Nanocarrier

Inorganic nanocarriers, including mesoporous silica nanoparticles [89], metal–organic frameworks [90], metal oxide nanoparticles [91], and mineral-based nanomaterials [92], have attracted considerable interest as versatile platforms for cancer therapy because of their high structural stability, tunable surface chemistry, substantial drug-loading capacity, and intrinsic physicochemical functionalities [93,94,95]. These systems may also provide catalytic activity [96], imaging capabilities [97,98], and stimuli-responsive therapeutic functions [99], thereby facilitating bufalin delivery and enabling multifunctional antitumour applications.

**Figure 3:**
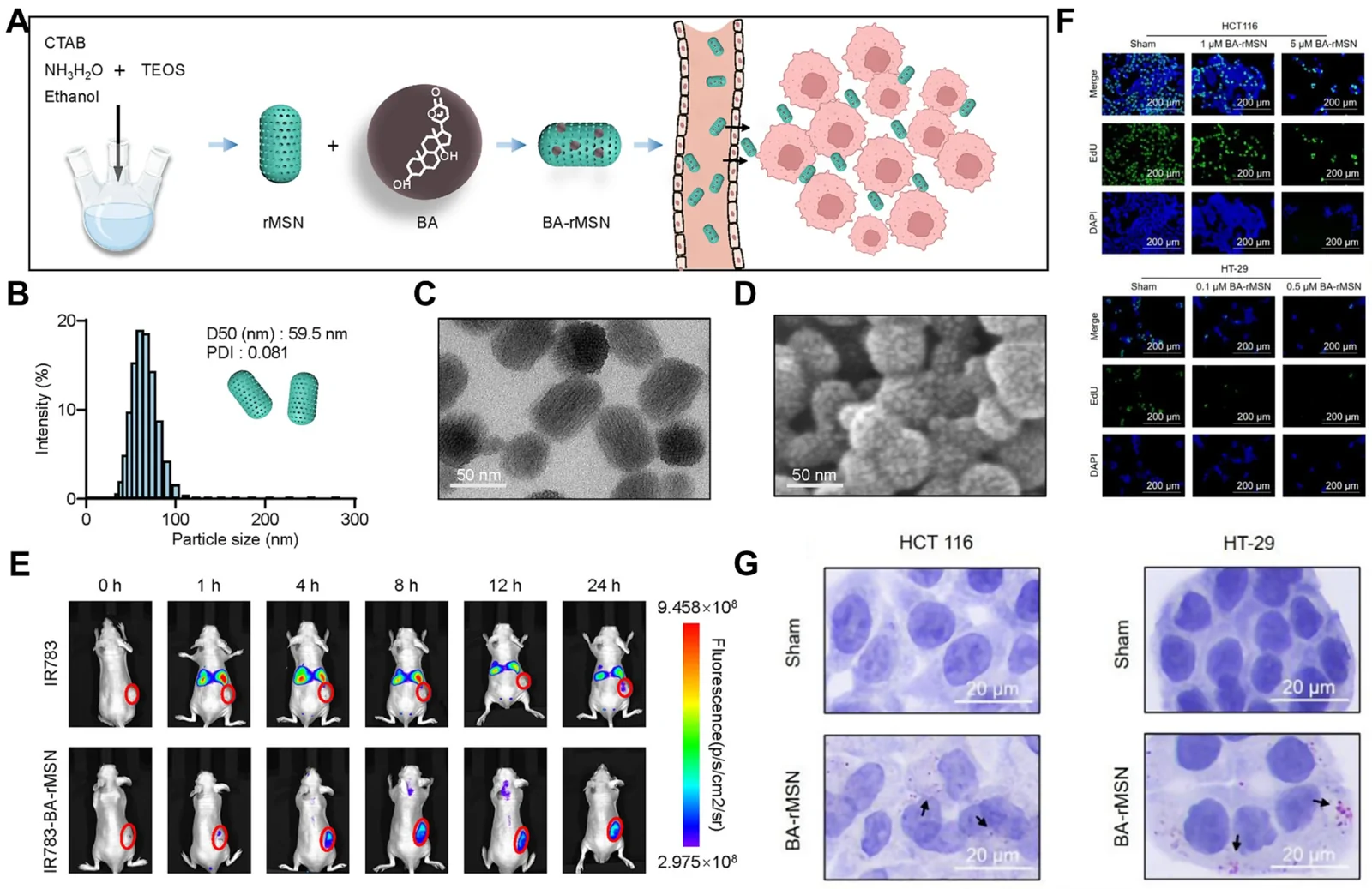
Application of organic nanocarriers loaded with bufalin in cancer therapy. (**A**) rMSNs were synthesized using CTAB, NH_3_H_2_O, and TEOS; BA was then loaded into the rMSNs. (**B**) The BA-rMSNs exhibited a consistent particle size distribution, as shown by their median diameter (D50) of 59.5 nm and polydispersity index (PDI) of 0.081. (**C**) The rod-shaped morphology of the BA-rMSNs was shown by TEM. (**D**) A rod-shaped, porous SEM picture. (**E**) In vivo fluorescence pictures demonstrated that the rMSN improved BA’s capacity to target tumours. (**F**) EdU quantitative analysis. (**G**) BA-rMSNs inhibited lipophagy using oil red O staining. Black arrows indicate representative areas with morphological alterations. Scale bars: 20 μm. Reproduced from reference [105]. Copyright 2024, Springer Nature.

Mesoporous silica nanoparticles (MSNs) are representative inorganic nanocarriers characterised by ordered pore structures, large specific surface areas, favourable physicochemical stability, and tunable drug-loading capacity. Their porous architecture facilitates the efficient encapsulation of hydrophobic molecules and enables controlled drug release [100,101,102]. Appropriate surface functionalisation may further improve colloidal stability, biocompatibility, and tumour accumulation [103,104]. Fan et al. [105] developed rod-shaped mesoporous silica nanoparticles loaded with bufalin (BA-rMSNs) to address the insufficient tumour exposure and cardiotoxicity associated with free bufalin (Figure 3A). The bufalin-loaded MSNs have excellent morphology, particle size, and polydispersity (Figure 3B–D). The rod-like MSN framework enabled efficient bufalin encapsulation and sustained drug release under tumour-relevant acidic conditions, resulting in enhanced tumour accumulation and reduced cardiotoxicity in vivo (Figure 3E). BA-rMSNs markedly suppressed colorectal cancer-cell proliferation and induced apoptosis by disrupting lipid-droplet metabolism. Mechanistically, the nanoplatform inhibited lipophagic flux, as indicated by the accumulation of LC3 and p62, and activated mechanistic target of rapamycin (mTOR) signalling. These alterations disrupted lipid homeostasis and contributed to the suppression of tumour growth (Figure 3F,G).

Overall, inorganic nanocarriers represent promising platforms for bufalin delivery because of their structural stability, high drug-loading capacity, and controlled-release capabilities. Among these platforms, mesoporous silica-based systems have demonstrated the potential to enhance tumour accumulation, reduce bufalin-associated toxicity, and improve antitumour efficacy by modulating tumour-relevant biological processes. These findings suggest that inorganic nanoplatforms may serve as effective delivery systems for expanding the therapeutic utility of bufalin.

### 4.3. Biomimetic Nanocarriers

Biomimetic nanocarriers, including cell membrane-coated nanoparticles [106,107] and extracellular vesicle-mimetic nanovesicles [108,109], represent promising platforms for cancer therapy by integrating natural biological interfaces with synthetic nanomaterials. These systems retain biological functions associated with immune evasion [110], prolonged systemic circulation [111], and tumour-selective targeting [112], thereby improving drug-delivery efficiency and therapeutic precision in bufalin-based cancer treatment.

**Figure 4:**
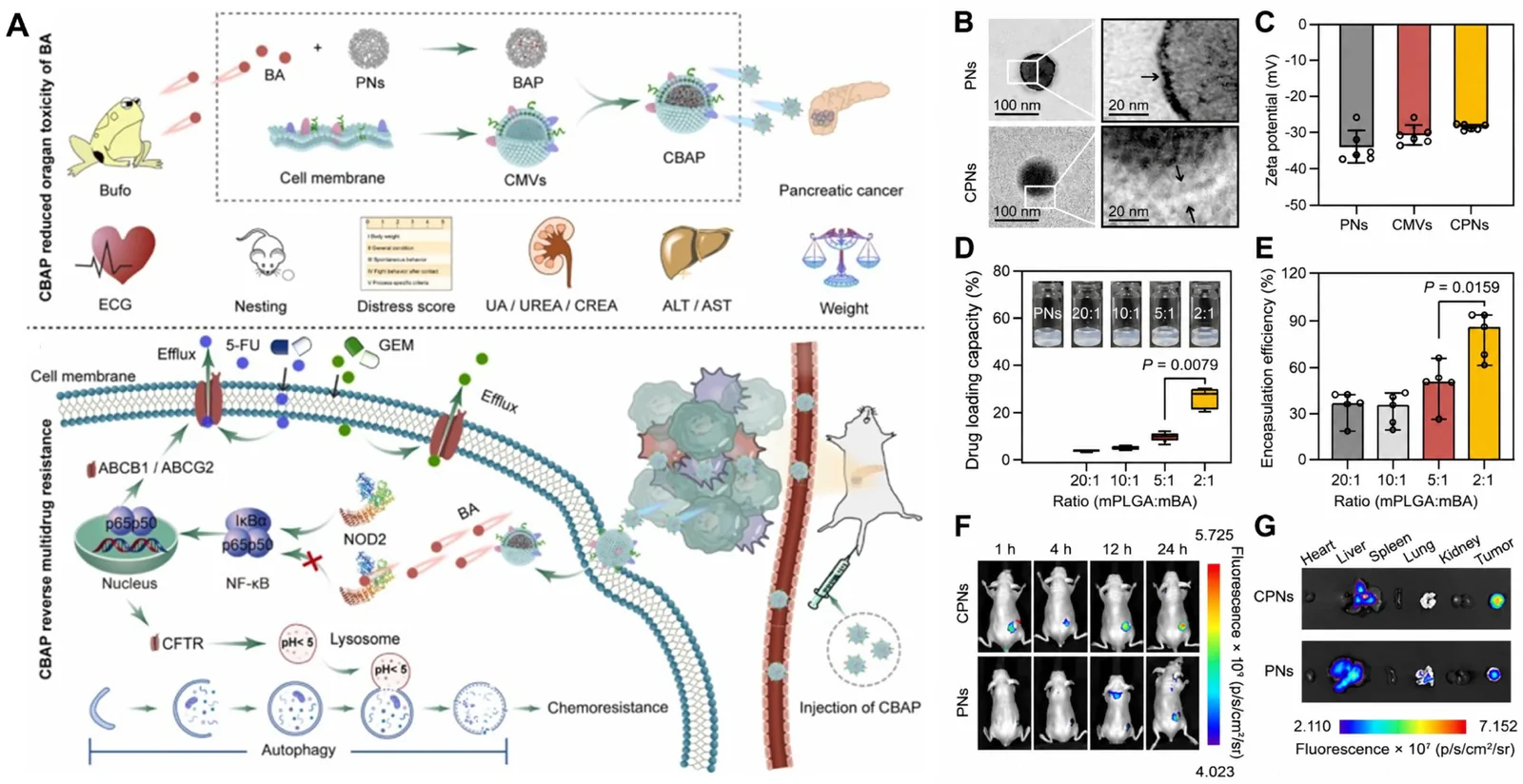
Application of biomimetic nanocarriers loaded with bufalin in cancer therapy. (**A**) CBAP successfully overcomes multidrug resistance in pancreatic cancer and lessens the organ toxicity of BA. To create CBAP, we encapsulated BA in cell membrane-camouflaged PLGA nanoparticles; the mice’s heart, liver, and kidneys were unaffected by CBAP. Through the NOD2/NF-κB/ABC transporter axis, CBAP altered chemoresistance by directly targeting NOD2. TEM (**B**) and CPN zeta potential (**C**). (**D**) Drug loading capacity and (**E**) CBAP encapsulation effectiveness. (**F**) CPNs are better than PNs in delivering BA to tumour locations, as shown by in vivo fluorescent pictures and ex vivo results (**G**). Reproduced from reference [119]. Copyright 2023, Elsevier Ltd.

Camouflaged cell membrane nanoparticles are an important class of biomimetic nanoplatforms prepared by coating synthetic nanoparticle cores with natural cell membranes [113]. The design synergistically merges the structural versatility of synthetic nanomaterials with membrane-derived functions such as immune evasion, extended systemic circulation and tumour-targeted delivery. From isolated membranes derived from erythrocytes, tumour cells, immune cells or combinations of different cell membranes, these platforms can be built [114,115,116,117]. The retention of membrane proteins and surface receptors may endow these nanoparticles with biomimetic recognition properties and enhance their accumulation within tumours [118]. Zhang et al. [119] designed a hybrid membrane-coated nanoparticle for bufotalin delivery, a bufadienolide structurally similar to bufalin, in colorectal cancer (Figure 4A). The nanoparticle was coated with a hybrid membrane of erythrocytes and cancer cells. The component of the erythrocyte membrane decreased immune recognition and increased systemic circulation, while the component of the cancer-cell membrane increased tumour accumulation via homotypic recognition. The morphology, zeta potential, encapsulation efficiency and drug loading capacity of the bufalin-loaded biomimetic nanomedicine were excellent (Figure 4B–E). The nanoplatform enabled controlled bufotalin release and enhanced intracellular drug accumulation, thereby improving antitumour efficacy through the integration of chemotherapy and photothermal therapy (Figure 4F,G). Mechanistically, the system increased reactive oxygen species production, suppressed HIF-1α signalling, downregulated glutathione peroxidase 4 (GPX4) expression, and promoted ferroptosis. These effects contributed to tumour-growth inhibition while reducing systemic toxicity.

In short, biomimetic nanocarriers provide a distinctive strategy for improving bufalin delivery by integrating synthetic nanoplatforms with natural biological interfaces. Through cell membrane camouflage and biomimetic recognition, these systems may enhance tumour accumulation, evade immune clearance, and increase intratumoural drug exposure. These properties may strengthen bufalin-mediated anticancer activity while reducing off-target toxicity.

### 4.4. Hybrid Nanocarriers

Hybrid nanocarriers integrate two or more organic, inorganic, carbon-based, or biomimetic components within a single delivery platform. By exploiting the complementary properties of their constituent materials, these platforms can simultaneously improve the aqueous solubility, loading efficiency, formulation stability, tumour accumulation, and stimuli-responsive release of bufalin while also enabling diagnostic imaging and multimodal therapy. Their modular architectures further allow bufalin-based chemotherapy to be integrated with ion-interference therapy, ferroptosis induction, phototherapy, gene-editing strategies, and immunomodulation, while reducing premature drug release, systemic exposure, and off-target toxicity.

#### 4.4.1. Organic–Inorganic Hybrid Nanocarriers

**Figure 5:**
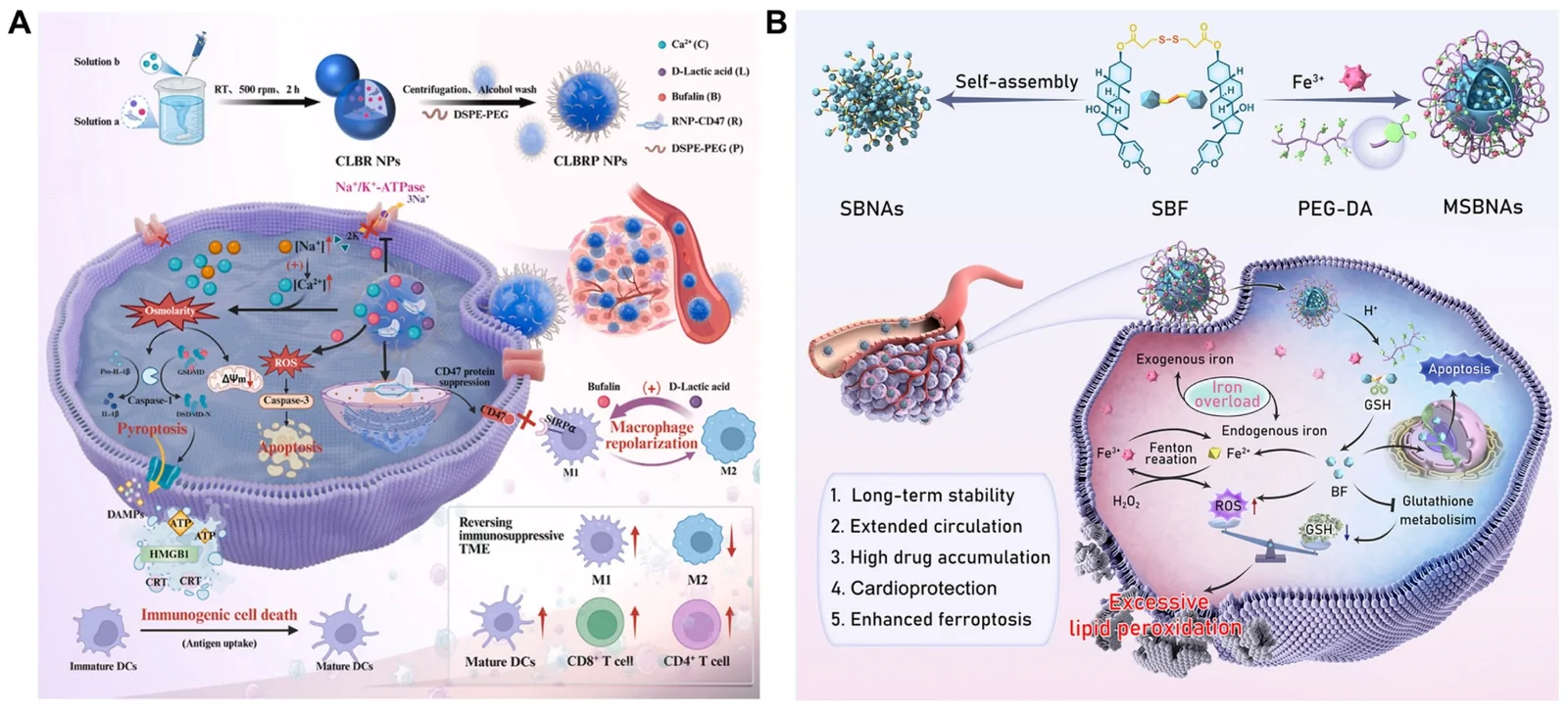
Application of organic–inorganic hybrid nanocarriers loaded with bufalin in cancer therapy. (**A**) When CLBRP releases its payload intracellularly, it increases intracellular osmotic pressure, induces pyroptosis, apoptosis, and immunogenic cell death, improves macrophage phagocytic efficiency, and synergistically promotes anticancer treatment. Reproduced from reference [124]. Copyright 2025, Elsevier Ltd. (**B**) Schematic diagram of the preparation of MSBNAs as well as their therapeutic mechanism. Reproduced from reference [128]. Copyright 2025, Elsevier Ltd.

Organic–inorganic hybrid nanocarriers combine functional inorganic materials with organic drugs and polymeric matrices, thereby integrating stimuli-responsive degradation, sustained drug release, and therapeutic ion delivery within a single platform [120,121,122]. These systems may comprise an inorganic mineral phase, an organic drug-containing component, and a biodegradable hydrogel matrix designed to enhance local retention and reduce premature systemic exposure. To achieve localised and sustained bufalin delivery, Li et al. [123] embedded calcium carbonate nanoparticles within an alginate hydrogel matrix to fabricate a bufalin-loaded calcium carbonate nanoparticle–hydrogel system (BCNPs@gel). In this hybrid nanoplatform, CaCO_3_ nanoparticles served as acid-responsive inorganic components that released bufalin and Ca^2+^ within the acidic tumour microenvironment, whereas the alginate hydrogel prolonged local retention and regulated drug diffusion. Following intratumoural administration, BCNPs@gel gradually released bufalin, which inhibited Na^+^/K^+^-ATPase activity and promoted intracellular Na^+^ accumulation. The resulting elevation in intracellular Na^+^ reversed Na^+^/Ca^2+^ exchange, leading to Ca^2+^ overload and mitochondrial dysfunction. This Ca^2+^ overload triggered GSDME-dependent pyroptosis, accompanied by the release of damage-associated molecular patterns and the induction of immunogenic cell death. In addition, the released Ca^2+^ promoted dendritic-cell maturation and cytotoxic T-cell infiltration, thereby enhancing antitumour immune activation. When combined with anti PD-1 therapy, BCNPs@gel remodelled the immunosuppressive tumour microenvironment and enhanced systemic antitumour immunity, resulting in marked inhibition of melanoma progression. This study illustrates how an organic–inorganic hybrid nanoplatform can convert bufalin-induced ion dysregulation into pyroptosis-mediated cancer immunotherapy. In addition, Liu et al. [124] constructed pH-responsive CLBRP nanoparticles by co-precipitating calcium hydroxide and D-lactic acid in the presence of bufalin and CD47-targeting CRISPR-Cas9 ribonucleoproteins, followed by surface modification with DSPE-PEG (Figure 5A). Under acidic conditions, the nanoparticles released more than 80% of their bufalin and Cas9/sgRNA payloads, together with Ca^2+^. The released bufalin induced reactive oxygen species-dependent apoptosis and inhibited Na^+^/K^+^-ATPase, thereby increasing intracellular Na^+^ and Ca^2+^ levels, disrupting osmotic and mitochondrial homeostasis, and activating caspase-1/GSDMD-dependent pyroptosis. The resulting release of adenosine triphosphate (ATP), high-mobility group box 1 (HMGB1), interleukin-1β (IL-1β), and interleukin-18 (IL-18), together with calreticulin exposure, promoted immunogenic cell death and dendritic-cell maturation. Meanwhile, bufalin and D-lactate repolarised M2-like macrophages towards an M1-like phenotype, whereas CRISPR-mediated CD47 disruption attenuated the tumour-cell “don’t eat me” signal and enhanced macrophage-mediated phagocytosis. Consequently, CLBRP increased CD8^+^ T-cell infiltration, suppressed both primary and rechallenge tumours, reduced pulmonary metastasis, and alleviated bufalin-associated cardiotoxicity. Another important class of organic–inorganic hybrid nanocarriers is based on metal–phenolic networks, which combine metal-ion-mediated therapeutic functions with the high drug-loading capacity and tumour responsiveness of organic prodrug assemblies [125,126,127]. This integration can improve structural stability, pharmacokinetic behaviour, and therapeutic selectivity. These systems are typically assembled from metal ions, polyphenolic ligands, PEG-containing stabilisers, and stimuli-cleavable organic prodrugs. Wu et al. [128] developed metal-phenolic-network-driven bufalin homodimeric prodrug nano-coassemblies (MSBNAs) by assembling the disulfide-linked prodrug BF-SS-BF with PEG-functionalised polyphenols and Fe^3+^ (Figure 5B). The approximately 150 nm core–shell nanoparticles achieved a bufalin encapsulation efficiency of 59.68% and were cooperatively stabilised by hydrophobic interactions, electrostatic interactions, and metal-phenolic coordination. MSBNAs remained stable under physiological conditions but disassembled in acidic, glutathione-rich tumour environments. Under these conditions, disruption of metal–phenolic coordination and cleavage of disulfide bonds resulted in the release of more than 70% of the bufalin payload. The nanoplatform prolonged systemic circulation, enhanced tumour penetration and accumulation, and markedly reduced cardiac drug exposure. Mechanistically, bufalin increased reactive oxygen species levels, depleted glutathione, and suppressed SLC7A11 and GPX4 expression, whereas MPN-derived Fe^3+^ supplied exogenous iron and promoted NCOA4-associated intracellular iron accumulation. This simultaneous disruption of antioxidant defence and iron homeostasis amplified lipid peroxidation and ferroptotic cell death. Consequently, MSBNAs achieved a tumour-growth inhibition rate of 83.74% and almost completely suppressed pulmonary metastasis in osteosarcoma xenograft models, without causing evident cardiotoxicity. Additionally, Shang et al. [129] developed bufalin-loaded Fe_3_O_4_@PDA-PEG-cRGD nanoparticles for magnetic resonance imaging-guided mild photothermal therapy of colorectal cancer. Bufalin was adsorbed onto the polydopamine shell through non-covalent intermolecular interactions, whereas cRGD promoted integrin αvβ3-mediated tumour-cell uptake. Acidic conditions and 808 nm laser irradiation accelerated bufalin release, whereas Fe_3_O_4_ and polydopamine enabled T_2_-weighted magnetic resonance imaging and efficient photothermal conversion. Mechanistically, the released bufalin suppressed SRC-3/HIF-1α signalling, downregulated GLUT1 and LDHA expression, and reduced glucose uptake, lactate production, and ATP generation. The consequent reduction in ATP-dependent HSP70 expression attenuated tumour thermoresistance, thereby enhancing apoptosis and achieving a tumour-growth inhibition rate of 84.63% under mild photothermal treatment.

#### 4.4.2. Inorganic–Inorganic Hybrid Nanocarriers

**Figure 6:**
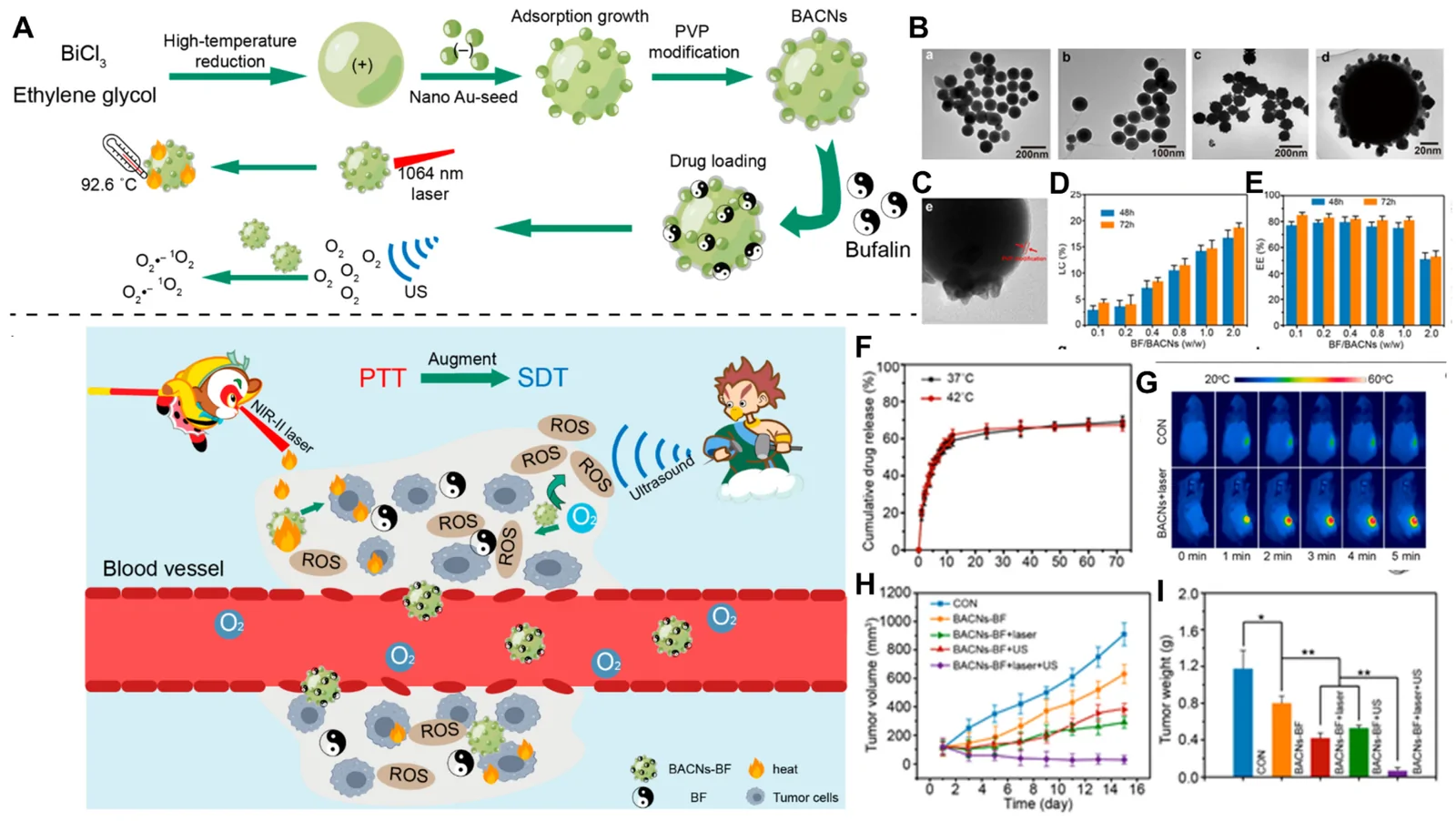
Application of inorganic–inorganic hybrid nanocarriers loaded with bufalin in cancer therapy. (**A**) A schematic representation of the main steps in the synthesis of multifunctional BACN drug loading, release at the tumour, and synergistic PTT (eyes of the monkey king)/SDT (thunder hammer of thunder king) antitumor are all part of the BACNs-BF antitumor mechanism. (**B**) Structure characterization of BACNs. (**a**,**b**) TEM images of Bi NPs. (**c**–**e**) TEM images of BACNs. (**C**) BACN TEM images; (**D**) BACNs-BF LC and (**E**) EE values over 48 and 72 h. (**F**) BACNs-BF drug release patterns in PBS were investigated in vitro at various temperatures. (**G**) NIR pictures of SMMC-7721 tumour mice taken every minute. Tumour volume curves (**H**) and tumour weights (**I**) of mice with SMMC-7721 tumours. * *p* < 0.05, ** *p* < 0.01. Reproduced from reference [136]. Copyright 2023, American Chemical Society.

Metal-based hybrid nanocomposites represent an emerging class of inorganic hybrid nanoplatforms that integrate multiple metallic components with complementary physicochemical properties, including catalytic activity, photothermal conversion, and energy-responsive therapeutic functions [130,131,132]. These systems are typically constructed from functional metallic nanostructures, such as gold- [133], bismuth- [134], or iron-based materials [135], together with other inorganic components whose distinctive optical, catalytic, and energy-conversion properties can support multimodal cancer therapy. Zhang et al. [136] developed gold-crowned bismuth-based nanocomposites by decorating bismuth-based nanoparticles with gold nanostructures (Figure 6A). The nanocomposites exhibited excellent nanomedicine properties and strong stability (Figure 6B–F**)**. The resulting multifunctional platform integrated sonodynamic therapy, photothermal therapy, and chemotherapy. The gold component enhanced near-infrared light absorption and photothermal conversion, whereas the bismuth-based component promoted ultrasound-triggered reactive oxygen species generation for sonodynamic therapy (Figure 6G). Upon external stimulation, the nanocomposites induced local hyperthermia and oxidative stress, while the incorporated chemotherapeutic agents provided an additional cytotoxic effect. The combined sonodynamic therapy, photothermal therapy, and chemotherapy strategy induced pronounced mitochondrial dysfunction, intracellular oxidative damage, and apoptotic cell death, resulting in greater tumour suppression than that achieved with any single treatment modality (Figure 6H,I). This study highlights the potential of metal-based hybrid nanocomposites as multifunctional therapeutic platforms that integrate energy conversion, catalytic activity, and drug-mediated tumour cytotoxicity.

#### 4.4.3. Organic–Organic Hybrid Nanocarriers

**Figure 7:**
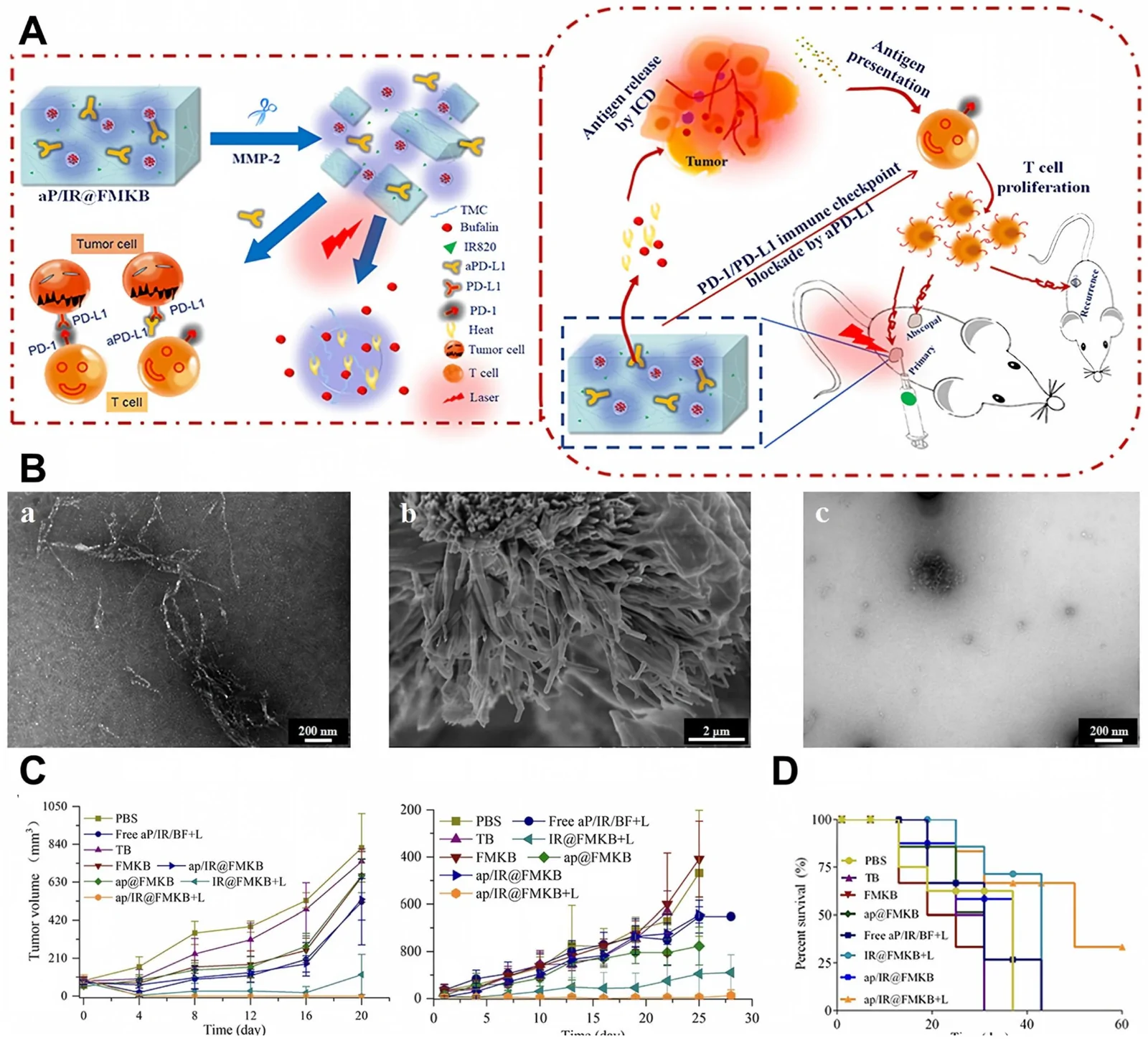
Application of organic–organic hybrid nanocarriers loaded with bufalin in cancer therapy. (**A**) Schematic illustration of drug release and tumour inhibition of the intratumoural-injected aP/IR@FMKB hydrogel. (**B**) (**a**) TEM (Scale bar = 200 nm), and (**b**) SEM (Scale bar = 2 μm) images of aP/IR@FMKB hydrogel; (**c**) TEM (Scale bar, 200 nm) examination of aP/IR@FMKB hydrogel after incubating with MMP-2 enzyme. (**C**) The main and recurring tumours undergoing various treatments (three minutes of laser irradiation at a photodensity of 0.5 W/cm^2^). The data were shown as mean ± SD (n = 6). (**D**) The 4T1 recurrent tumour’s survival rate. All of the mice in the various groups died at the following times: PBS, day 37; TB NPs, day 31; FMKB, day 31; aP@FMKB, day 31; Free aP/IR/BF + L, day 43; IR@FMKB + L, day 43; aP/IR@FMKB, day 37; aP/IR@FMKB + L, day 60. Reproduced from reference [138]. Copyright 2023, Taylor & Francis.

Organic–organic hybrid nanocarriers integrate multiple organic components within a single multifunctional platform, enabling coordinated drug delivery and tumour-microenvironment modulation for synergistic cancer therapy [137]. Zhang et al. [138] reported on an MMP-2-responsive peptide hydrogel-based hybrid nanoplatform, termed aP/IR@FMKB. The system integrated bufalin-loaded trimethyl chitosan nanoparticles, the photothermal agent IR820, and an anti PD-L1 antibody within a self-assembled peptide hydrogel. The system incorporated MMP-2-cleavable peptide sequences and exploited CRGDK-mediated tumour penetration to enable local drug release and enhance intratumoural accumulation. Under near-infrared laser irradiation, IR820 generated a photothermal effect that enhanced bufalin-mediated immunogenic cell death (ICD), as evidenced by calreticulin exposure and the release of ATP and HMGB1. Concurrent anti-PD-L1 blockade enhanced CD8^+^ T-cell infiltration and activation, thereby contributing to the remodelling of the immunosuppressive tumour microenvironment. This hybrid platform significantly inhibited primary, recurrent and distal tumours in 4T1 breast cancer models, highlighting the potential of organic hybrid nanocarriers for multimodal cancer immunotherapy. Another important type of organic–organic hybrid nanocarrier is the combination of thermosensitive liposomes and polymeric hydrogels, which combines the high drug encapsulation capacity of lipid vesicles with the local retention, injectability and stimuli-responsive release properties of hydrogels [139,140,141]. Such systems usually consist of thermoresponsive phospholipids, PEGylated lipids, biodegradable block copolymers and photothermal agents, which enable in situ gelation and externally controlled drug release. Wang et al. [142] developed an injectable, photothermally responsive composite hydrogel, termed Bu&Ap-Lip@IR820 Gel, for the prevention of postoperative colon cancer recurrence (Figure 7A). Bufalin and apatinib were co-encapsulated within thermosensitive liposomes composed of DPPC, MSPC, and DSPE-PEG_2000_. The drug-loaded liposomes and IR820 were subsequently incorporated into a PLGA-PEG-PLGA hydrogel (Figure 7B). Following injection into the surgical tumour bed, the formulation underwent rapid gelation at physiological temperature, resulting in prolonged local drug retention and reduced premature drug diffusion. Under 808 nm laser irradiation, IR820 induced local hyperthermia, promoted the gel-to-sol transition and liposomal membrane permeabilisation, and thereby accelerated the release of bufalin and apatinib. The combined treatment induced apoptosis and inhibited tumour-cell proliferation, migration, invasion, and angiogenesis. These effects were accompanied by increased cleaved caspase-3 levels and reduced expression of Ki-67, MMP-2, VEGF, and CD31. In a postoperative HCT-116 tumour model, the treatment achieved a recurrence-inhibition rate of 66.67% and exhibited favourable biocompatibility (Figure 7C,D).

#### 4.4.4. Organic–Inorganic-Biomimetic Hybrid Nanocarriers

**Figure 8:**
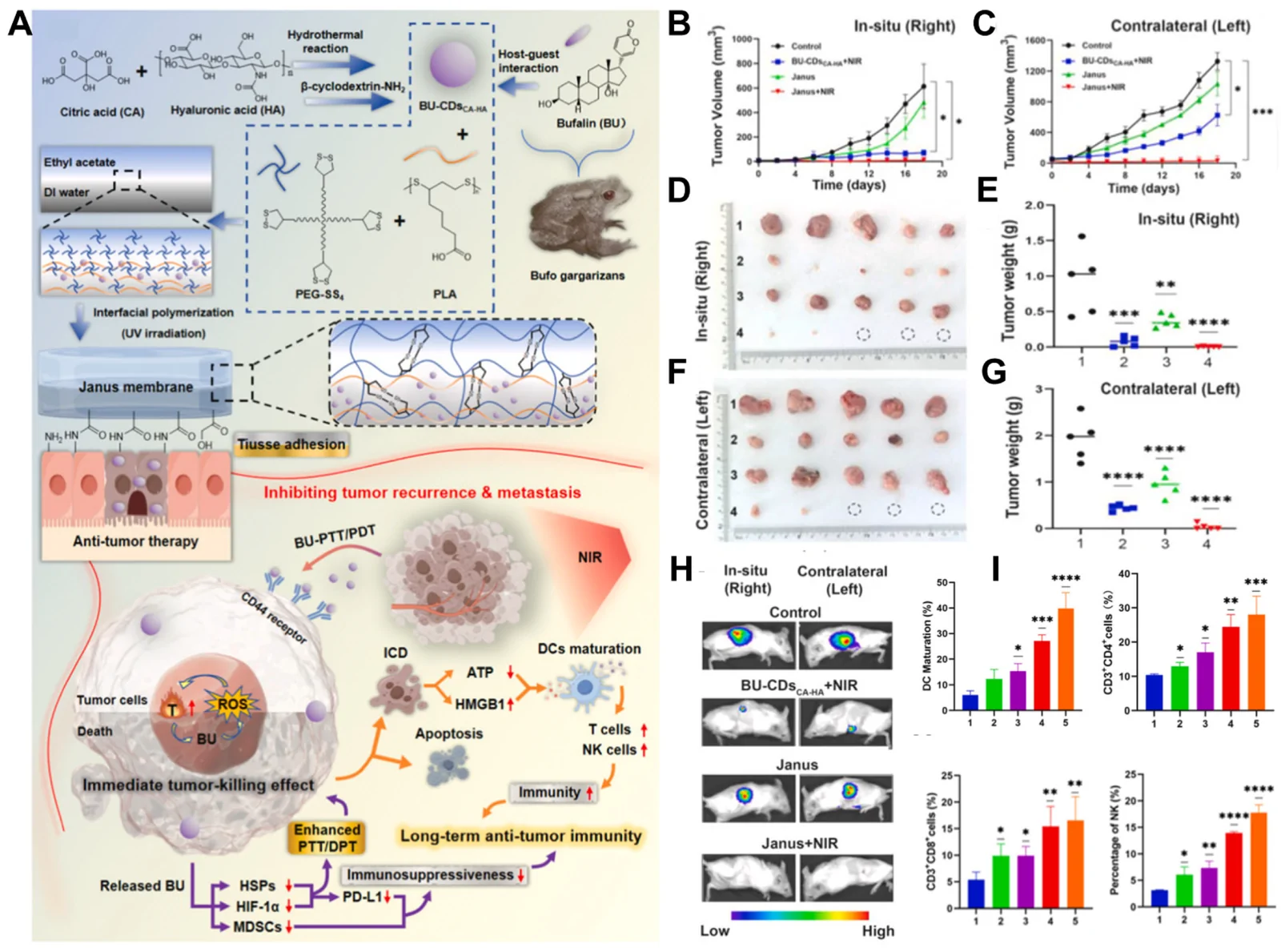
Application of organic–inorganic-biomimetic hybrid nanocarriers loaded with bufalin in cancer therapy. (**A**) The Janus membrane and BU-CDsCA-HA preparation process; (**B**) the BU-CDsCA-HA-loaded Janus membrane was linked to the tumour via NIR, bringing PTT and PDT, as part of the treatment process for CRC tumours. Meanwhile, BU-CDsCA-HA selectively infiltrated tumour cells after being liberated from the Janus membrane. Through BU, heat, and ROS, the BU-PTT/PDT combination had an instantaneous tumour-killing impact by causing ICD in tumour cells and thus triggering an anti-tumour immune response. Additionally, BU may lessen TCM’s immunosuppression, promoting a sustained anti-tumour immune response. The combined offense–defence approach may improve CRC tumour inhibition and stop metastasis and recurrence. (**B**,**C**) In situ and contralateral tumour volume changes during the course of treatment, * *p* < 0.05, *** *p* < 0.001. (**D**–**G**) Following the mice’s euthanasia, the gross observation and weight analysis of the in situ and contralateral tumours of all groups showed * *p* < 0.05, ** *p* < 0.01, *** *p* < 0.001, and **** *p* < 0.0001. (**H**) In situ and contralateral tumour imaging on day eighteen. (**I**) Quantitative analysis and flow cytometry of DCs, CD4^+^/CD8^+^ T cells, and NK cells, *** *p* < 0.001, **** *p* < 0.0001. Reproduced from reference [147]. Copyright 2026, Elsevier Ltd.

Carbon-based biomimetic hybrid nanocarriers integrate photoactive carbon nanomaterials with biologically inspired targeting functions and asymmetric interfacial architectures, thereby enabling local retention, controlled drug release, tissue protection, and multimodal cancer therapy [143,144,145,146]. These systems commonly incorporate carbon dots, receptor-targeting biomacromolecules, host–guest drug-loading units, and tissue-adhesive polymer networks. Yu et al. [147] developed a bufalin-loaded phototherapeutic Janus membrane by incorporating citric acid-derived, hyaluronic acid-modified carbon dots (BU-CDs_CA-HA_) into a bilayer composed of four-arm disulphide-functionalised polyethylene glycol and poly(lipoic acid) (Figure 8A). β-Cyclodextrin immobilised on the carbon dot-encapsulated bufalin through host–guest interactions, achieving an encapsulation efficiency of 91.2%. The Janus membrane adhered firmly to the postoperative tumour bed and reduced the initial 24 h burst release from approximately 80% to 47%. Under 808 nm laser irradiation, the carbon dots simultaneously generated heat and reactive oxygen species, enabling combined photothermal and photodynamic tumour destruction. Bufalin further downregulated HSP70, HSP90, HIF-1α, and PD-L1 expression, thereby attenuating phototherapy resistance and tumour immune evasion (Figure 8B–G). The combined treatment induced immunogenic cell death, promoted dendritic-cell maturation, activated CD4^+^ and CD8^+^ T cells and natural killer cells, and reduced the abundance of myeloid-derived suppressor cells (Figure 8H,I). These immune effects were associated with marked suppression of local recurrence and distant metastasis in postoperative colorectal cancer models.

Collectively, the studies reviewed here indicate that hybridisation enables individual material components to perform distinct yet coordinated functions in bufalin delivery and cancer treatment. Inorganic components can provide therapeutic ion release, imaging, catalytic activity, and energy conversion; organic matrices regulate drug loading and release; and biomimetic interfaces improve localisation and biological interactions. Such functional integration may transform bufalin from a poorly deliverable cytotoxic compound into a programmable therapeutic component of multimodal and immune-responsive anticancer nanomedicine. Table 1 summarizes various studies on bufalin-loaded nanodelivery systems in cancer therapy.

## 5. Challenges and Future Perspectives

Despite encouraging preclinical findings, the development of bufalin-based nanomedicines remains at an early stage of clinical translation. The principal challenge is not a lack of therapeutic functionality but the translation of increasingly sophisticated formulations into pharmaceutical products with well-defined identities, reproducible quality, predictable in vivo behaviour, and a demonstrable improvement in therapeutic index [148].

First, bufalin has a narrow therapeutic window, and nanocarrier-mediated tumour accumulation does not, by itself, guarantee an acceptable safety profile. Future studies should establish quantitative relationships among the administered dose, released and unbound bufalin, active metabolites, tissue exposure, pharmacodynamic responses, and cardiotoxicity. Measurements of total bufalin concentration or bulk elemental content alone are insufficient when hybrid systems contain prodrugs, metal ions, degradable matrices, or multiple therapeutic cargos. Chemical speciation, unbound and carrier-associated fractions, degradation products, and organ-specific retention should therefore be evaluated through exposure-informed pharmacological studies and repeated-dose toxicity testing [149].

In addition, material complexity must be mechanistically justified. Many platforms combine targeting ligands, photothermal agents, immune-checkpoint inhibitors, metal ions, hydrogels, and responsive linkers; however, the contribution of each component is frequently inferred rather than directly demonstrated. Future development should adhere to the principle of minimum sufficient complexity. Factorial experimental designs, pathway inhibition, gene knockout, immune-cell depletion, rescue experiments, and appropriate monotherapy controls are required to distinguish genuine therapeutic synergy from additive toxicity or formulation-dependent changes in drug exposure [150].

Importantly, greater attention should be paid to the dynamic structural evolution of bufalin nanomedicines in biological fluids. Protein adsorption, ligand exchange, enzymatic degradation, oxidation, and competition with endogenous biomolecules may alter nanoparticle integrity and surface identity before the carrier reaches the tumour. Consequently, physicochemical and biological characterisation should extend beyond the initial assessment of particle size and morphology to include serum stability, protein-corona composition, release kinetics, carrier disassembly, intracellular trafficking, and the pharmacological activity of transformation products [151].

More notably, clinical indications should be selected on the basis of their biological compatibility with the delivery platform rather than broad claims of tumour targeting. Potential predictors of treatment response include Na^+^/K^+^-ATPase expression, SRC-3/HIF-1α activity, redox vulnerability, ferroptosis sensitivity, CD44 and integrin expression, immune-checkpoint status, and tumour accessibility. Locoregional and externally activated approaches may offer more practical routes to early clinical translation. Examples include postoperative tumour-bed treatment, superficial phototherapy, intratumoural administration, and image-guided intervention. These approaches may initially be more feasible than complex multifunctional formulations designed for systemic administration [152].

Finally, successful translation will require scalable manufacturing processes, validated potency assays, long-term storage stability, and clinically relevant experimental models. Patient-derived organoids, immunocompetent tumour models, metastatic and recurrence models, and large-animal studies should complement conventional subcutaneous xenograft models. Ultimately, a bufalin nanomedicine should advance towards clinical development only when it offers a clear advantage over free bufalin or simpler formulations in terms of safety, dosing convenience, patient selection, durability of therapeutic response, or treatment monitoring. The future of the field therefore lies not in maximising nanoplatform complexity but in establishing a precise correspondence among bufalin pharmacology, material function, tumour biology, and clinically measurable benefit.

## 6. Conclusions

In summary, bufalin represents a promising anticancer agent with diverse pharmacological activities, including the degradation of oncogenic proteins, induction of regulated cell death, metabolic reprogramming, and modulation of the tumour microenvironment. Furthermore, bufalin-based nanodelivery systems can enhance therapeutic potential by addressing tumour accumulation, enabling controlled release, and increasing the efficacy of combination therapies. Advances in tumour biology and materials science and the comprehensive exploration of natural products are expected to accelerate the development of bufalin-based nanomedicines toward clinical translation.

## Figures and Tables

**Table 1:** Bufalin-loaded nanodelivery systems in cancer therapy.

Specific Nanocarrier Type	Delivery Platform and Cargo	Cancer Therapy Strategy	Experimental Models and Major Anticancer Mechanisms	Safety Evaluation	Ref.
HA-functionalized fusogenic pH-sensitive liposome	pSL-cocktail (Bufalin; Doxorubicin)	Chemotherapy + Cancer stem-cell-targeted therapy	In vitro/ex vivo: HER2-positive BT474 and HCC1954 breast cancer cells; homotypic/heterotypic spheroids; patient-derived organoids. In vivo: orthotopic HCC1954 xenografts in BALB/c nude mice. Mechanism: eliminates CD44-high cancer stem cells, suppressing proliferation, mammosphere formation, self-renewal, migration, and invasive dissemination.	In vitro: normal human dermal fibroblasts were included in formulation/cytotoxicity assessment. In vivo: pSL-cocktail was well tolerated; no weight loss or obvious toxic signs were reported in liposomal groups, and H&E analysis showed no major-organ tissue damage. Free-drug cocktail caused severe systemic toxicity (3/6 deaths after the first injection and acute tonic-clonic seizures in survivors).	[61]
RGD-functionalized VES-g-CSO/TPGS mixed micelle	BU@MMs (Bufalin)	Chemotherapy	In vitro: A2780 and SKOV3 ovarian cancer cells. In vivo: ID8 intraperitoneal metastasis model in female C57BL/6 mice. Mechanism: induces mitochondrial apoptosis and reverses EMT: E-cadherin increases; N-cadherin, vimentin, Snail, MMP-2, MMP-9, and VEGF decrease.	In vivo: no evident body-weight difference versus free bufalin; BU@MMs caused almost no damage to vital organs, including kidneys, and showed better biocompatibility/biosafety than free bufalin.	[69]
pH-sensitive liposome	Buf/Dox-CDpSL (Bufalin; Doxorubicin)	Chemotherapy + Breast cancer stem-cell eradication	In vitro only: trastuzumab-sensitive BT-474 and trastuzumab-resistant BT-474R HER2-positive breast cancer cells, including ALDH-positive BCSC assays and mammosphere/self-renewal models. Mechanism: synergistically eliminates ALDH-positive breast cancer stem cells, reducing mammosphere formation, tumorigenesis, self-renewal, and bulk-cell viability.	No dedicated in vivo safety study was reported in this article. No normal-cell or animal safety endpoint sufficient for a comparative safety conclusion was identified.	[77]
Rod-shaped mesoporous silica nanoparticle	BA-rMSNs (Bufalin)	Chemotherapy	In vitro: HCT116 and HT-29 colon cancer cells. In vivo: HCT116 subcutaneous xenografts in male BALB/c nude mice. Mechanism: blocks lipophagy through mTOR activation, causing LC3/p62 accumulation, lipid-droplet retention, proliferation inhibition, and cell death.	In vivo cardiotoxicity: ECG plus serum CK, α-HBDH and LDH were evaluated after BA or BA-rMSN treatment. BA-rMSNs reduced bufalin-mediated cardiotoxicity compared with free bufalin.	[94]
Cancer-cell-membrane-camouflaged PLGA nanoparticle	CBAP (Bufalin; Gemcitabine, 5-fluorouracil)	Chemotherapy	In vitro: PANC-1, SW1990, MIA PaCa-2, CFPAC-1, SUIT-2, GEM-MIA PaCa-2 and GEM-PANC-1 pancreatic cancer models. In vivo: MIA PaCa-2 orthotopic pancreatic xenografts and additional xenograft studies in male BALB/c nude mice. Mechanism: targets NOD2/NF-κB, downregulates ABCB1/ABCG2, blocks autophagic flux, reverses multidrug resistance, and enhances chemotherapy-induced apoptosis and pyroptosis.	In vivo: toxicity was assessed by ECG, body weight, distress score and nesting behaviour. CBAP markedly mitigated acute bufalin cardiotoxicity; combination therapy reduced tumour burden without significant body-weight or nesting-score deterioration.	[108]
pH-responsive CaCO_3_ nanoparticle–alginate hydrogel	BCNPs@gel (Bufalin; Anti-PD-1 antibody)	Chemotherapy + Immunotherapy	In vitro: B16F10 melanoma cells. In vivo: B16F10 tumour-bearing C57 mice. Mechanism: Na^+^/K^+^-ATPase inhibition reverses NCX, causing Ca^2+^ overload, caspase-3/GSDME pyroptosis, ICD, dendritic-cell maturation, and CD8^+^ T-cell activation.	In vivo: no sudden body-mass loss was observed with BCNPs@gel; main-organ H&E sections appeared normal. In contrast, free bufalin caused acute toxicity (seizure/dizziness associated with arrhythmia) and treatment-related deaths. BCNPs@gel showed no obvious toxicity or accidental death.	[112]
DSPE-PEG-modified calcium lactate nanoparticle	CLBRP NPs (Bufalin; Cas9/sgRNA-CD47 ribonucleoprotein)	Chemotherapy + CRISPR gene therapy + Apoptosis/pyroptosis + Immunotherapy	In vitro: CT26 colorectal cancer cells; macrophage-polarization/phagocytosis experiments. In vivo: subcutaneous CT26 tumours in BALB/c mice, plus tumour-rechallenge and experimental lung-metastasis models. Mechanism: Ca^2+^-overload pyroptosis and apoptosis induce ICD; CD47 editing enhances macrophage phagocytosis, M1 polarization, CD8^+^ infiltration, and immune memory.	In vivo: CLBRP did not cause significant body-weight loss or cardiac abnormalities. H&E showed no abnormalities in heart, liver, spleen, lung or kidney; serum TP, ALB, ALT, AST, ALP and BUN were not significantly different from PBS controls.	[113]
Metal–phenolic-network-driven homodimeric prodrug nano-coassembly	MSBNAs (Bufalin; Fe^3+^)	Chemotherapy + Ferroptosis	In vitro: 143B and SJSA-1 osteosarcoma cells. In vivo: osteosarcoma CDX and PDX mouse models, including lung-metastasis evaluation. Mechanism: iron overload and oxidative stress suppress SLC7A11/GPX4 and increase NCOA4, lipid peroxidation, and ferroptotic tumour-cell death.	In vivo: MSBNAs avoided the body-weight loss seen with free bufalin and preserved cardiac function on echocardiography. Histopathology showed markedly reduced cardiomyocyte injury/apoptosis; CDX/PDX studies reported no significant body-weight loss or major-organ histopathological abnormalities.	[117]
cRGD-targeted Fe_3_O_4_@PDA magnetic photothermal nanoparticle	Fe_3_O_4_@PDA-PEG-cRGD/BU NPs (Bufalin)	Chemotherapy + Photothermal therapy	In vitro: HCT116 colorectal cancer cells. In vivo: HCT116 tumour-bearing BALB/c nude mice. Mechanism: suppresses SRC-3/HIF-1α-GLUT1/LDHA signalling, depletes ATP and HSP70, inhibits glycolysis, and sensitizes tumours to PTT.	In vivo: body weight remained stable; H&E of heart, liver, spleen, lung and kidney showed no evident pathological changes; serum ALT, AST, BUN and creatinine remained within normal ranges, indicating no evident systemic toxicity at the tested dose.	[118]
Gold-crowned bismuth hybrid nanocomposite	BACNs-BF (Bufalin)	Chemotherapy + Sonodynamic therapy + Photothermal therapy	In vitro: SMMC-7721 and HCC-LM3 liver cancer cells and 4T1 breast cancer cells. In vivo: SMMC-7721 subcutaneous tumour model in BALB/c mice. Mechanism: ultrasound-generated ROS and NIR-II hyperthermia synergize with bufalin, increasing Bax, decreasing Bcl-2, and inducing mitochondrial apoptosis.	In vitro: unloaded BACNs showed no apparent cytotoxicity in the tested cancer cells at concentrations up to 400 μg/mL. In vivo: routine blood/biochemistry indices after BACNs-BF were within normal ranges, and H&E showed no organ or inflammatory damage in heart, liver, spleen, kidney or lung.	[125]
MMP-2-responsive tumour-penetrating peptide hydrogel	aP/IR@FMKB (Bufalin; IR820; Anti-PD-L1 antibody)	Chemotherapy + Photothermal therapy + Immunotherapy	In vitro: 4T1 breast cancer cells and 4T1 multicellular spheroids; ICD assays. In vivo: 4T1 primary, recurrent and bilateral/distal tumour models in female BALB/c mice. Mechanism: MMP-2-triggered release enables deep penetration; PTT and bufalin induce ICD, sensitizing PD-L1 blockade and activating dendritic/CD8^+^ T cells.	In vitro: blank hydrogel maintained >90% viability in 4T1, NIH 3T3 and HC11 cells. In vivo: no significant weight loss; H&E showed no visible inflammation or tissue injury in heart, liver, spleen or kidney.	[127]
Thermosensitive liposome–PLGA-PEG-PLGA hydrogel	Bu&Ap-Lip@IR820 Gel (Bufalin; Apatinib; IR820)	Chemotherapy + Photothermal therapy	In vitro: HCT116 colorectal cancer cells; HUVEC angiogenesis assays; NCM460 normal colon epithelial cells for compatibility. In vivo: postoperative HCT116-Luc recurrence model in female BALB/c nude mice. Mechanism: NIR-triggered release combines bufalin cytotoxicity, apatinib antiangiogenesis, and PTT; caspase-3 increases, whereas Ki-67, MMP-2, VEGF, and CD31 decrease.	In vitro: hydrogel extracts were assessed in NCM460 cells; hemolysis rates of Gel, Lip Gel and Lip@IR820 Gel were 0.47%, 3.63% and 3.93% (<5%). In vivo: no weight loss; major-organ H&E and blood biochemical indices showed no significant treatment-related abnormalities or organ damage.	[131]
BU-loaded carbon-dot Janus membrane (organic–inorganic–biomimetic local platform)	BU-CDs_CA-HA_-Janus (Bufalin)	Chemotherapy + Photothermal therapy + Photodynamic therapy + Immunotherapy	In vitro: CT26 and HCT116 colorectal cancer cells; RAW264.7 macrophages; ICD/DC-related assays. In vivo: CT26 subcutaneous tumour model and postoperative local-recurrence/distal-metastasis CRC models in BALB/c mice. Mechanism: PTT/PDT induces ICD; bufalin suppresses HSP70/HSP90/HIF-1α/PD-L1, activates dendritic, T, and NK cells, and reduces MDSCs.	In vitro: β-CDsCA-HA/Janus material maintained >90% viability in CT26, HCT116, RAW264.7 and FHC cells; no obvious hemolysis was observed. In vivo: subcutaneous membrane degradation caused no obvious skin necrosis, oedema or inflammation; body weight remained stable and major-organ H&E showed no physiological morphological abnormalities. The Janus membrane also reduced local thermal injury by insulating adjacent skin.	[136]

Abbreviations: 5-FU, 5-fluorouracil; ALB, albumin; ALDH, aldehyde dehydrogenase; ALP, alkaline phosphatase; ALT, alanine aminotransferase; AST, aspartate aminotransferase; BA/BF/BU, bufalin; BCSC, breast cancer stem cell; BUN, blood urea nitrogen; CDX, cell-derived xenograft; CK, creatine kinase; CRC, colorectal cancer; CSC, cancer stem cell; DC, dendritic cell; DOX, doxorubicin; ECG, electrocardiography; EMT, epithelial–mesenchymal transition; FOLFIRINOX, folinic acid/5-fluorouracil/irinotecan/oxaliplatin; GSH, glutathione; H&E, haematoxylin and eosin; HIF-1α, hypoxia-inducible factor-1α; HSP, heat shock protein; ICD, immunogenic cell death; LDH, lactate dehydrogenase; MMP, matrix metalloproteinase; MPN, metal–phenolic network; MRI, magnetic resonance imaging; NCX, Na^+^/Ca^2+^ exchanger; NIR, near-infrared; NP, nanoparticle; PBS, phosphate-buffered saline; PDA, polydopamine; PDT, photodynamic therapy; PDX, patient-derived xenograft; PEG, polyethylene glycol; PLGA, poly(lactic-co-glycolic acid); PTT, photothermal therapy; RGD, Arg-Gly-Asp; RNP, ribonucleoprotein; ROS, reactive oxygen species; SDT, sonodynamic therapy; TP, total protein; TPGS, D-α-tocopheryl polyethylene glycol 1000 succinate; VEGF, vascular endothelial growth factor; VES-g-CSO, vitamin E succinate-grafted chitosan oligosaccharide; α-HBDH, α-hydroxybutyrate dehydrogenase.

## Data Availability

No new data were created or analysed in this study. Data sharing is not applicable to this article.
